# Copper-Induced Thyroid Disruption and Oxidative Stress in *Schizopygopsis younghusbandi* Larvae

**DOI:** 10.3390/antiox15010112

**Published:** 2026-01-15

**Authors:** Liqiao Zhong, Chi Zhang, Fei Liu, Haitao Gao, Dengyan Di, Fan Yao, Baoshan Ma, Mingdian Liu, Xinbin Duan

**Affiliations:** 1 National Agricultural Science Observing and Experimental Station of Chongqing, Yangtze River Fisheries Research Institute, Chinese Academy of Fishery Sciences, Wuhan 430223, China; zhonglq@yfi.ac.cn (L.Z.); yaofann@stu.scu.edu.cn (F.Y.); baoshanma@yfi.ac.cn (B.M.); 2 Institute of Fisheries Science, Xizang Academy of Agricultural and Animal Husbandry Sciences, Lhasa 850000, China; zc0891@163.com (C.Z.); liufei636@163.com (F.L.); 3 Yunnan Academy of Fishery Sciences, Kunming 655000, China; ynkhtt@126.com; 4 Bureau of Agriculture and Rural Affairs of Gongshan County, Nujiang 673599, China; dengyandi22@163.com; 5 College of Fisheries, Huazhong Agricultural University, Wuhan 430070, China; 6 College of Water Resource & Hydropower, Sichuan University, Chengdu 610065, China

**Keywords:** Cu^2+^, *Schizopygopsis younghusbandi*, thyroid disruption, antioxidant stress, developmental toxicity

## Abstract

In recent years, heavy metal emissions in Lhasa have been increasing, which has an impact on the local water environment. The negative effects of copper (Cu^2+^) on aquatic ecosystems have attracted much attention, as even low concentrations of Cu^2+^ can exert toxic effects on aquatic organisms. However, the impact of Cu^2+^ on native fish species from the Lhasa River remains poorly understood. In this study, *Schizopygopsis younghusbandi* (*S. younghusbandi*) larvae were exposed to Cu^2+^ at concentrations of 0. 5, 5, 50, and 500 μg/L for 7 or 14 days to evaluate its toxic effects on thyroid function and the antioxidant system. The results indicate that whole-body total thyroxine (T4) and triiodothyronine (T3) levels were significantly decreased following Cu^2+^ exposure. This decrease was accompanied by a marked increase in *dio1* and *dio2* gene expression and decreased expression of thyroid hormone synthesis genes (*nis*, *tg*, *ttf1* and *pax8*) after exposure to Cu^2+^. Furthermore, the activity of superoxide dismutase (SOD), catalase (CAT), and glutathione reductase (GR) and the content of lipid peroxidation were increased, while the content of glutathione (GSH) was decreased. In addition, the survival rates and body lengths of *S. younghusbandi* larvae were significantly reduced following 7- and 14-day Cu^2+^ exposure. The Integrated Assessment of Biomarker Response (IBR) analysis further revealed dose- and time-dependent effects of Cu^2+^ on the larvae. In conclusion, the findings demonstrate that Cu^2+^ exposure induced disruption of thyroid endocrine and antioxidant systems and caused developmental toxicity in *S. younghusbandi* larvae.

## 1. Introduction

As the largest tributary of the Brahmaputra River, the Lhasa River plays a crucial role in the Lhasa region by providing the main water source for drinking, agriculture, and industry. The exploration of geothermal and mineral resources, along with municipal waste discharge, has introduced heavy metals into the rivers [1,2,3]. The Tibetan Plateau acts as a regional convergence zone for airborne contaminants, potentially leading to the accumulation of pollutants [4]. Previous studies indicated that the health risk caused by heavy metals (containing As, Cd, Cu, Zn, and Pb) in the Lhasa River has exceeded the standard values recommended by the Environmental Protection Agency [5]. A recent study showed that the content of Cu^2+^ in water samples of the Lhasa River reached up to 15.4 μg/L, exceeding class III of the Chinese surface water quality standard [6]. Cu^2+^ has also been detected in native fish from the Lhasa River, including *Ptychobarbus dipogon*, *Schizopygopsis stoliczkae*, *Schizopygopsis younhusbandi* (*S. younghusbandi*), *Schizopygopsis microphalus,* and *Oxygymnocypris stewartia*; the highest Cu^2+^ concentration in the liver reached 59 μg/g wet weight [7]. Among heavy metals (including As, Cd, Cr, Cu, Hg, Mn, Pb, and Zn), Cu^2+^ poses the highest ecological risk to aquatic organisms in the Lhasa River [8]. Thus, there might be undiscovered safety hazards related to Cu^2+^ pollution in the Lhasa River basin.

Cu^2+^ is a vital trace element required by living organisms [9,10]. It plays an important role as an enzymatic cofactor in energy production, iron and oxygen transport, metabolism, hormone processing, and signal transduction [11]. Nevertheless, higher concentrations of this essential heavy metal can cause negative effects on organisms [12]. Exposure to Cu^2+^ induced multiple adverse effects in fish, including physiological, behavioral, and immunological alterations, histomorphological damage to gills, and growth performance impairment [13]. Additionally, previous studies showed that Cu^2+^ induced developmental toxicity and oxidative stress in fish [14,15]. Cu^2+^ is also an endocrine-disrupting heavy metal [16]; the hypothalamic–pituitary–gonadal (HPG) axis was disrupted in Cu^2+^-exposed zebrafish [16]. The results from our recent study indicated that Cu^2+^ induced thyroid-disrupting effects in zebrafish [17]. Although various effects of Cu^2+^ on fish have been well documented, its impact on the native fish species of the Lhasa River remains limited.

Cu^2+^ poses the highest ecological risk to aquatic organisms, and despite its well-known adverse effects on fish, the specific effects of Cu^2+^ on the native fish species inhabiting the Lhasa River remain poorly understood. Additionally, fish residing in alpine lakes or rivers are more susceptible to pollutants [18]. *S. younghusbandi* emerges as the predominant fish species within the Lhasa River, accounting for 20–40% of total fishery catch [19,20]. It also serves as a key indicator species within the specific aquatic ecosystem [21]. Consequently, *S. younghusbandi* serves as an excellent model for investigating the responses of various environmental pollutants. The thyroid hormone (TH) system is essential for regulating fish growth, development, and metabolism [22], making it a sensitive target for endocrine disruption. As mentioned above, Cu^2+^ has the ability to interfere with the hypothalamus–pituitary–thyroid (HPT) axis and induce oxidative stress. Therefore, it is necessary to assess the effects of Cu^2+^ on the TH system and oxidative stress in *S. younghusbandi*.

In the present study, *S. younghusbandi* larvae were exposed to Cu^2+^ at different concentrations for 7 or 14 days with the aim of evaluating its toxic effects on thyroid endocrine and antioxidant systems. The aims of our study were as follows: (1) to verify whether Cu^2+^ can disturb the thyroid endocrine and antioxidant systems of *S. younghusbandi* and (2) to assess the disruptive effects of Cu^2+^ on *S. younghusbandi* and elucidate the potential mechanisms.

## 2. Materials and Methods

### 2.1. Chemicals and Reagents

CuSO_4_ (purity ≥ 99%) was purchased from Aladdin (Shanghai, China) and was subsequently dissolved in deionized water. TRIzol reagent was obtained from Invitrogen (Carlsbad, CA, USA). Total thyroid hormone (TH) levels, including thyroxine (T4) and triiodothyronine (T3), were quantified using specific enzyme-linked immunosorbent assay (ELISA) kits (T3: CEA453Ge; T4: CEA452Ge) purchased from Cloud-Clone Corp (Katy, TX, USA). The activities of superoxide dismutase (SOD, A001-1-1), catalase (CAT, A007-1-1), and glutathione reductase (GR, A062-1-1) and the contents of glutathione (GSH, A006-2-1) and malondialdehyde (MDA, A003-1-1) were determined using commercial assay kits from the Nanjing Jiancheng Bioengineering Institute (Nanjing, China), strictly following the manufacturer’s protocols. All reagents employed in this study were of analytical grade.

### 2.2. Larvae Culture and Exposure

*S. younghusbandi* larvae were provided by the Xizang Academy of Agriculture and Animal Husbandry Sciences. This work was approved by the Animal Experimental Ethical Inspection of Laboratory Animal Centre, Yangtze River Fisheries Research Institute, Chinese Academy of Fishery Sciences (No. 20190415001). Due to the absence of an established rearing protocol for this species, larvae were maintained in Holt buffer (3.5 g/L NaCl, 0.05 g/L KCl, 0.025 g/L NaHCO_3_, 0.1 g/L CaCl_2_, pH = 8.76, DO = 6.89 mg/L) for 7 days prior to the exposure experiment. Preliminary observations indicated that the survival rates in the Holt buffer exceeded 98%. Then, healthy larvae at 10 days post fertilization (10 dpf) were maintained in Holt buffer at a controlled temperature (12 ± 0.5 °C), with a 12:12 (light/dark) photoperiod. The larvae were not fed during the exposure periods, as the experiment was conducted entirely during their endogenous yolk sac stage, before they started exogenous feeding at about 25 dpf. A total of 200 larvae per replicate were randomly selected and transferred into glass beakers containing 500 mL exposure solutions (0, 0.5, 5, 50, and 500 μg/L Cu^2+^) for either 7 days (17 dpf) or 14 days (24 dpf). This resulted in an initial density of 0.4 larvae/mL. Three replicates were performed for each group. The glass beakers were covered with perforated plastic lids to minimize evaporation and contamination. Gentle and continuous aeration was provided to maintain dissolved oxygen. As Cu^2+^ concentrations in surface waters had been reported to range from 0.5 to 1000 μg/L [23], the chosen Cu^2+^ concentrations were environmentally relevant. Exposure was conducted in a semi-static system with complete daily renewal of the exposure medium. Key water quality parameters (pH, DO, and temperature) were monitored daily throughout the exposure period to ensure stability. The measured ranges were as follows: pH 8.70–8.82, DO 6.72–6.95 mg/L, and temperature 12.0 ± 0.5 °C. To verify that the Cu^2+^ concentrations matched the nominal levels, water samples were collected within 2 h of solution renewal and analyzed following a previously established method [24]. The measured Cu^2+^ concentrations in the treatment groups were maintained within 82.7% to 106.4% of the nominal values. As the larvae were in the yolk sac stage and not fed, and the exposure medium was completely renewed daily, and parameters such as ammonia, nitrite, and nitrate were not monitored. During the experimental period, dead larvae were removed daily. Developmental indices including survival rate, malformation frequency, and body length were recorded at the end of the exposure periods (7 and 14 days). Survival rate was calculated as the percentage of live larvae relative to the initial number. Malformation frequency was expressed as the percentage of larvae exhibiting any morphological abnormality. Body length was measured from the tip of the head to the end of the tail using a calibrated micrometer under a stereomicroscope. Subsequently, larvae from each group (varied exposure duration) were randomly selected, promptly flash-frozen in liquid nitrogen, and stored at −80 °C for analysis of gene expression and TH levels.

### 2.3. Gene Expression Analysis

To further determine gene expression in *S. younghusbandi* larvae, six pooled samples (n = 3 larvae per pool) were randomly selected. Total RNA was extracted, purified, quantified, and reverse-transcribed into first-strand cDNA, followed by mRNA expression analysis, all conducted according to established protocols described in previous studies [25,26]. UltraSYBR Mixture (CWBIO, Beijing, China, CW2602M) was used for quantitative RT-PCR (qRTPCR), which was then analyzed on an ABI 7500 System (Applied Biosystems, Foster City, CA, USA). Gene expression levels were calculated using the 2^−ΔΔCt^ method [27]. The qRT-PCR protocol consisted of an initial denaturation step at 95 °C for 10 min, followed by 40 cycles of 95 °C for 15 s and 60 °C for 1 min. The amplification efficiencies of all primer sets ranged from 95% to 105%. Detailed sequences of the quantitative qRT-PCR primers are provided in Table 1. Serving as a housekeeping gene, *β-actin* was stably expressed regardless of Cu^2+^ exposure. The average Ct values of *β-actin* were 16.68 ± 0.39 and 16.57 ± 0.45 following 7 and 14 days of exposure, respectively. Expression levels of target genes were normalized to *β-actin* mRNA to ensure accurate quantification.

### 2.4. TH Assay

Following 7 or 14 days of Cu^2+^ exposure, *S. younghusbandi* larvae from each group were collected for TH analysis. Detection of T4 and T3 levels was carried out according to a previously described method [25]. Briefly, approximately 100 larvae from each of the three replicate exposure beakers (n = 3) were pooled separately and homogenized on ice in phosphate-buffered saline (PBS) using a glass grinder. The homogenates were then centrifuged at 2348× *g* for 30 min at 4 °C, and the resulting supernatant was used to quantify TH levels via ELISA kits (Cloud-Clone Corp, Katy, TX, USA). Briefly, standards and samples were added to the pre-coated plate, followed by the addition of the detection antibody and enzyme conjugate. After incubation and washing, the substrate solution was added for color development. The reaction was stopped, and the absorbance was measured at 450 nm. The detection limits for T4 and T3 were 1.29 ng/mL and 51.7 pg/mL, respectively. According to the manufacturer’s specifications, the intra- and inter-assay coefficients of variation (CV) for the T3 and T4 ELISA kits were <10% and <12%, respectively.

### 2.5. Assay of Biochemical Parameters

After 7 or 14 days of Cu^2+^ exposure, *S. younghusbandi* larvae from each group were collected for antioxidant enzyme analysis. Frozen samples were thawed and homogenized on ice in 10 volumes of pre-chilled 0.86% physiological saline using an ultrasonic cell disrupter (Scientz-IID, Ningbo, China). The larval homogenate was centrifuged at 845× *g* for 10 min at 4 °C to provide the supernatant which was then used to measure the activities of CAT, SOD, and GR and the contents of GSH and MDA. The activities of SOD, CAT, and GR and the contents of GSH and MDA were determined using specific commercial assay kits purchased from the Nanjing Jiancheng Bioengineering Institute (Nanjing, China). The assays were performed strictly in accordance with the manufacturer’s protocols. The use of these kits for biochemical determination in tissue homogenates is well established and has been extensively documented in previous studies [28,29]. CAT activity was measured using the ammonium molybdate method. The reaction mixture contained the supernatant and substrate (H_2_O_2_) in phosphate buffer. After exactly 1 min of reaction at 37 °C, the reaction was terminated by the addition of ammonium molybdate. The residual H_2_O_2_ formed a stable yellow complex with molybdate, and its absorbance was measured at 405 nm. One unit of CAT activity was defined as the amount of enzyme that decomposes 1 μmol of H_2_O_2_ per second per mg of protein. The intra- and inter-assay CVs for the CAT activity assay kit were 1.9% and 4.94%, respectively. SOD activity was assessed using the hydroxylamine method (xanthine oxidase system). The assay system generates superoxide anions, which oxidize hydroxylamine to form nitrite. The nitrite then reacts with a chromogenic agent to produce a purple-red color, which was measured at 550 nm. SOD activity inhibits this color development. One unit of SOD activity was defined as the amount of enzyme that causes 50% inhibition of the nitrite formation reaction per mg of protein. According to the manufacturer’s specifications, the SOD activity assay kit demonstrated intra- and inter-assay CVs of 1.7% and 3.52%, respectively. GR activity was determined by monitoring the decrease in absorbance at 340 nm due to the oxidation of NADPH to NADP^+^. The reaction mixture contained the supernatant, oxidized glutathione (GSSG), and NADPH. The decrease in absorbance was recorded over a 2 min reaction interval. Activity was calculated using the extinction coefficient of NADPH and expressed as units per mg of protein, where one unit is defined as the amount of enzyme that oxidizes 1 μmol of NADPH per minute. The GR activity assay kit had reported intra- and inter-assay CVs of 2.5% and 4.25%, respectively. The GSH content was quantified using the 5,5′-dithiobis-(2-nitrobenzoic acid) (DTNB) method. The supernatant was deproteinized and then reacted with DTNB to form a yellow-colored 2-nitro-5-thiobenzoic acid (TNB), which was measured at 420 nm. GSH concentration was calculated against a standard curve of known GSH concentrations and expressed as μmol per mg of protein. For the GSH content assay, the kit’s intra- and inter-assay CVs were 1.2% and 3.86%, respectively. Similarly, MDA content was measured using the thiobarbituric acid (TBA) method. The supernatant was heated with TBA under acidic conditions (95 °C for 40 min) to form a pink MDA-TBA adduct. After cooling and centrifugation, the absorbance of the supernatant was read at 532 nm. MDA content was determined by comparing the absorbance to a standard curve prepared from tetraethoxypropane and expressed as nmol per mg of protein. The MDA content assay kit showed intra- and inter-assay CVs of 3.5% and 4.11%, respectively. Protein concentration was also determined using a commercial protein assay kit (Nanjing Jiancheng Bioengineering Institute).

### 2.6. Integrated Biomarker Response (IBR)

IBR combines multiple biomarkers into a single stress index, making it a valuable tool for evaluating ecological risk. The IBR was performed using the previously described method [30,31]. IBR version 2 was used in the present study. Biomarkers for IBR were selected based on their established roles in thyroid function and oxidative stress responses in fish. All detected biomarkers were standardized, with their scores shown in star plots.

### 2.7. Statistical Analysis

Data were presented as the mean ± standard deviation (SD). The normality of data distribution and homogeneity of variances were assessed using the Kolmogorov–Smirnov and Levene’s tests, respectively. When assumptions for parametric tests were violated, data were log-transformed. The transformed data were then re-tested for normality and homogeneity of variances. If the transformed data met the assumptions, a one-way analysis of variance (ANOVA) was performed. If the assumptions were still not satisfied after transformation, the non-parametric Kruskal–Wallis test was used. In this study, with the exception of the T3 data at 7 days, all transformed datasets met the parametric assumptions. As each gene and biochemical parameter was treated as an independent endpoint, multiple comparisons were controlled for within each analysis using Tukey’s test following ANOVA. The specific statistical tests applied to each variable were summarized in Supplementary Table S1. All statistical analyses were performed using SPSS 20.0 (IBM, Chicago, IL, USA), with a significance threshold set at *p* < 0.05.

## 3. Results

### 3.1. Developmental Toxicity

**Figure 1:**
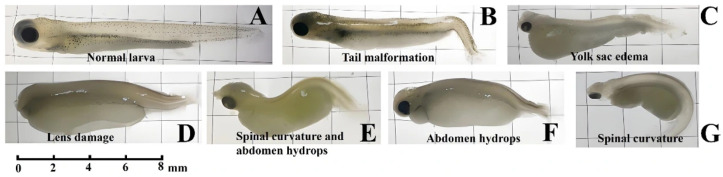
Morphology of *S. younghusbandi* larvae exposed to Cu^2+^. (**A**) Normal larva. (**B**) Tail malformation. (**C**) Yolk sac edema. (**D**) Lens damage. (**E**) Spinal curvature and abdomen hydrops. (**F**) Abdomen hydrops. (**G**) Spinal curvature.

Malformation rates exhibited an increasing trend following 7 and 14 days of Cu^2+^ exposure, with statistically significant increases (one-way ANOVA, Tukey’s test) observed in the 50 and 500 μg/L Cu^2+^ groups after 14 days. Cu^2+^ caused malformation in *S. younghusbandi* larvae including tail malformation (Figure 1B), yolk sac edema (Figure 1C), lens damage (Figure 1D), abdomen hydrops (Figure 1E,F), and spinal curvature (Figure 1E,G). Following 7 and 14 days of exposure, the survival rates in the Cu^2+^-treated groups showed a decreasing trend compared with the control group. After exposure for 7 days, there were no significant changes in all Cu^2+^ groups. After exposure for 14 days, the survival rate in the 50 and 500 μg/L Cu^2+^ groups were significantly lowered. After 7 and 14 days of exposure, the body length of larvae in the Cu^2+^-treated groups showed a decreasing trend compared with the control group, with the most pronounced reduction observed in the 500 μg/L Cu^2+^ exposure group (Table 2).

### 3.2. Gene Expression

**Figure 2:**
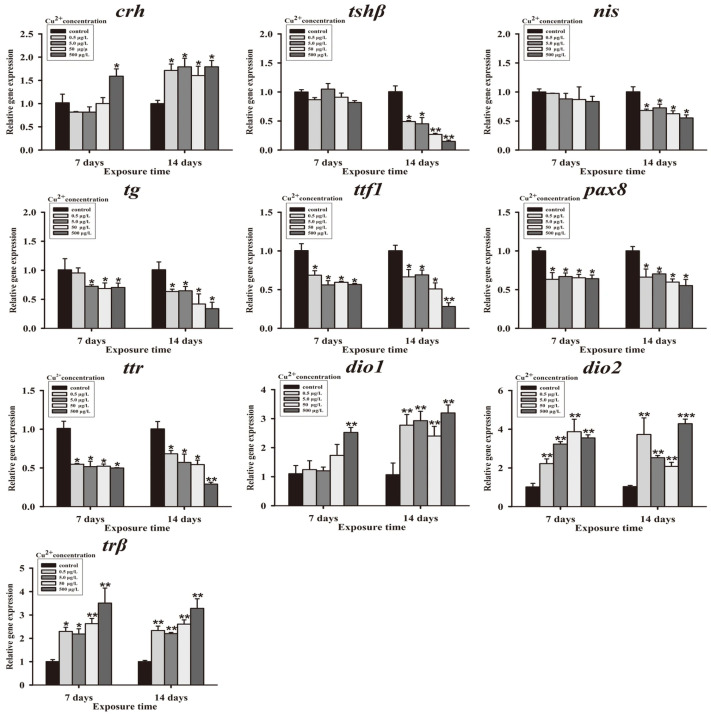
Gene, expression in *S. younghusbandi* larvae exposed to 0.5, 5, 50 and 500 μg/L Cu^2+^ for 7 days and 14 days. Data are presented as mean ± SD (n = 6). * *p* < 0.05, ** * p* < 0.01 and *** *p* < 0.001 indicate significant differences between exposure groups and corresponding control.

The expression of genes associated with the HPT axis in *S. younghusbandi* larvae was evaluated following 7 and 14 days of Cu^2+^ exposure. After 7 days, the expression levels of corticotropin-releasing hormone (*crh*) and type I iodothyronine deiodinase (*dio1*) were significantly upregulated 1.59- and 2.52-fold, respectively, in the 500 μg/L Cu^2+^ treatment group. Type II iodothyronine deiodinase (*dio2*) and TH receptor-β (*trβ*) genes were significantly increased in all Cu^2+^ exposure groups compared with the control group. However, exposure to 0.5, 5, 50, and 500 μg/L Cu^2+^ significantly downregulated mRNA expression of thyroid transcription factor-1 (*ttf1*), paired box protein 8 (*pax8*), and transthyretin (*ttr*) 0.69-, 0.56-, 0.60-, and 0.57-fold; 0.63-, 0.67-, 0.65-, and 0.64-fold; and 0.55-, 0.52-, 0.52-, and 0.50-fold, respectively. Meanwhile, the expression level of thyroid-stimulating hormone-β (*tshβ*) was not altered in all Cu^2+^ exposure groups (Figure 2).

After 14 days of exposure, mRNA expression of *crh* (1.71-, 1.80-, 1.60-, and 1.80-fold), *dio1* (2.78-, 2.93-, 2.40-, and 3.20-fold), *dio2* (3.73-, 2.53-, 2.08-, and 4.28-fold), and *trβ* (2.33-, 2.20-, 2.61-, and 3.29-fold) was significantly upregulated in all Cu^2+^ exposure groups. Moreover, mRNA expression of *tshβ* (0.55-, 0.52-, 0.52-, and 0.50-fold), *tg* (0.63-, 0.65-, 0.42-, and 0.34-fold), *ttf1* (0.66-, 0.69-, 0.51-, and 0.28-fold), *pax8* (0.66-, 0.70-, 0.60-, and 0.55-fold), and *ttr* (0.68-, 0.57-, 0.54-, and 0.30-fold) was significantly downregulated in all Cu^2+^ exposure groups (Figure 2).

### 3.3. TH Profile

**Figure 3:**
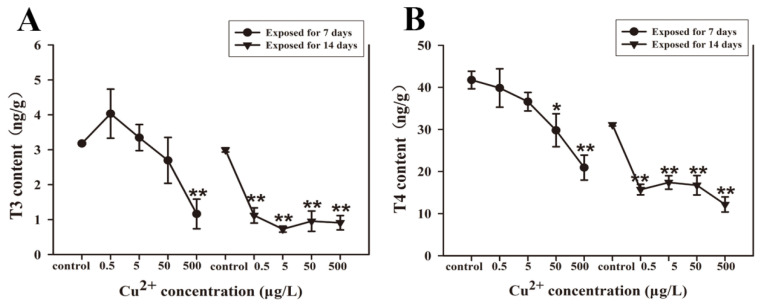
The whole-body total T3 (**A**) and T4 (**B**) content in *S. younghusbandi* larvae exposed to 0.5, 5, 50, and 500 μg/L Cu^2+^ for 7 days and 14 days. Data are presented as the mean ± SD (n = 3). * *p* < 0.05 and ** *p* < 0.01 indicate significant differences between the exposure groups and the corresponding control.

Whole-body total TH levels in the larvae were assessed following 7 and 14 days of Cu^2+^ exposure. After 7 days of exposure, T3 content was decreased significantly in the 500 μg/L Cu^2+^ exposure group compared with the control group (Figure 3A). Strikingly, T3 content was increased in the 0.5 and 5 μg/L Cu^2+^ exposure groups, though these increases were not statistically significant compared with the control group (Figure 3A). Moreover, compared with the control group (41.74 ng/g), whole-body total T4 (29.80 and 20.93 ng/g) contents were significantly decreased in the 50 and 500 μg/L Cu^2+^ exposure groups (Figure 3B). Compared with the control group (T3: 3.00 ng/g, T4: 31.12 ng/g), the whole-body total T3 (1.12, 0.73, 0.96, 0.91 ng/g) and T4 (15.72, 17.40, 16.74, 12.17 ng/g) contents were significantly decreased in all Cu^2+^ exposure groups after 14 days of exposure (Figure 3A,B).

### 3.4. Antioxidant and Detoxification Enzyme Activities

**Figure 4:**
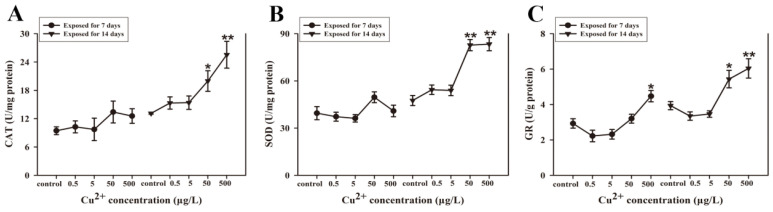
The levels of antioxidant enzyme activities (CAT (**A**), SOD (**B**), GR (**C**)) in *S. younghusbandi* larvae exposed to 0.5, 5, 50, and 500 μg/L Cu^2+^ for 7 days and 14 days. Data are presented as the mean ± SD (n = 3). * *p* < 0.05 and ** *p* < 0.01 indicate significant differences between the exposure groups and the corresponding control.

Figure 4 shows the impact of Cu^2+^ exposure on the activities of SOD, CAT, and GR in *S. younghusbandi* larvae. After 7 days of exposure, although CAT and SOD levels showed a slight increase across all Cu^2+^ treatment groups, these changes were not statistically significant (*p* > 0.05). However, CAT activity was significantly increased in the 50 and 500 μg/L Cu^2+^ exposure groups following 14 days of treatment. The percentage increase in CAT activity was 52.2% and 94.5%, respectively, compared with control (Figure 4A). Moreover, SOD activity was significantly increased in the 50 and 500 μg/L Cu^2+^ exposure groups following 14 days of treatment. SOD activities increased by 73.9% and 75.3% in the 50 and 500 μg/L Cu^2+^ exposure groups, respectively, compared with the control (Figure 4B). After 7 days of exposure, GR activity was significantly elevated in the 500 μg/L Cu^2+^ exposure group. After 14 days of exposure, GR activity levels were significantly elevated in both the 50 and 500 μg/L Cu^2+^ exposure groups, increasing by 37.8% and 53.3% in these respective treatment groups (Figure 4C).

### 3.5. Levels of Glutathione and Lipid Peroxidation

**Figure 5:**
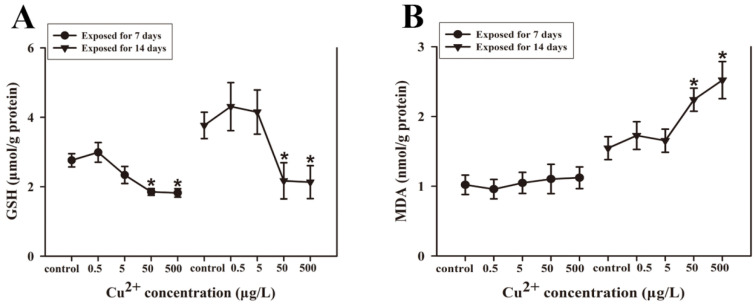
GSH (**A**) and MDA (**B**) contents in *S. younghusbandi* larvae exposed to 0.5, 5, 50, and 500 μg/L Cu^2+^ for 7 days and 14 days. Data are presented as mean ± SD (n = 3). * *p* < 0.05 indicate significant differences between exposure groups and corresponding control.

After 7 days of exposure, GSH levels were significantly decreased in the 50 and 500 μg/L Cu^2+^ groups, with reductions of 33.0% and 34.1%, respectively. Furthermore, GSH levels were decreased significantly in the 50 and 500 μg/L Cu^2+^ groups after 14 days of exposure. The percentage decrease in GSH levels were 42.4% and 43.5%, respectively. Of particular note, there were no significant changes in the 0.5 and 5 μg/L Cu^2+^ groups (*p* > 0.05) after 7 or 14 days (Figure 5A).

The levels of MDA in *S. younghusbandi* larvae are shown in Figure 5B. There were no significant changes in all Cu^2+^ exposure groups following 7 days of treatment (*p* > 0.05). Following 14 days of Cu^2+^ exposure, MDA levels rose considerably within the 0.5 and 5 μg/L Cu^2+^ groups. Additionally, GSH levels increased by 44.5% and 62.6% in these respective groups compared with the control (Figure 5B).

### 3.6. Integrated Assessment of Cu^2+^ Response

**Figure 6:**
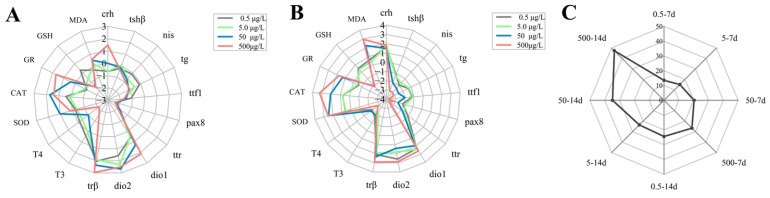
Star plots for biomarker responses in *S. younghusbandi* larvae after exposure to 0.5, 5, 50, and 500 μg/L Cu^2+^ for 7 days (**A**) and 14 days (**B**), and integrated biomarker response of all parameters measured after Cu^2+^ exposure (**C**).

The star plots (Figure 6A,B) present the parameters that characterize the reference deviation of each analyzed biomarker. Areas above 0 indicate biomarker induction, while areas below 0 correspond to biomarker inhibition. With the increase in concentration, the IBR values become higher (13.5, 15.1, 20.3, and 26.6 for 0.5, 5, 50, and 500 μg/L Cu^2+^, respectively) after larvae exposure to Cu^2+^ for 7 days. After 14 days of Cu^2+^ exposure, the IBR values are also increased (24.5, 23.6, 34.7, and 47.4 for 0.5, 5, 50, and 500 μg/L Cu^2+^ respectively) (Figure 6C).

## 4. Discussion

Cu^2+^ exposure induced significant developmental toxicity in *S. younghusbandi* larvae, as evidenced by reduced survival and body length, increased malformation rates, and various deformities including yolk sac edema, abdominal hydrops, and spinal curvature. Similar observations have been reported in previous studies, where Cu^2+^ exposure significantly impacted larval development by reducing survival rate, decreasing body length or weight, and increasing malformation rate [17,32]. In this study, whole-body total T4 and T3 levels in *S. younghusbandi* larvae were significantly reduced. THs are essential for regulating the development and growth of fish larvae [22]. The developmental toxicity in *S. younghusbandi* larvae exposed to Cu^2+^ may be attributed to impaired thyroid function.

In vertebrates, the hypothalamus releases thyroid-releasing hormone (TRH), which in turn stimulates the pituitary gland to release thyroid-stimulating hormone (TSH). TSH promotes iodine uptake by the thyroid gland and regulates the synthesis and secretion of THs [33]. In fish, TSH secretion is primarily regulated by CRH released from the hypothalamus. Previous studies have shown that THs influence the expression of *crh* in fish through a negative feedback loop [22]. It was reported that short-term Cu^2+^ exposure could reduce the levels of THs (T3 and T4) in Chinese rate minnow (*Gobiocypri rarus*) larvae, a change that is associated with increased expression of *crh* and *tshβ* [34]. In this study, we noted that the expression of *crh* was also increased in response to Cu^2+^ exposure for 7 days and 14 days, associated with the reduction in THs (T4 and T3). This outcome may be attributed to a negative feedback response triggered by the reduced TH levels in *S. younghusbandi* larvae. In vertebrates, TSH is encoded by the *tshβ* gene and regulates the thyroid axis by binding to its specific receptor on the thyroid gland [35].

PAX8 is critical for terminal follicular cell maturation, while TTF1 regulates thyroid ontogeny in fish [36,37]. The observed downregulation of *ttf1* and *pax8* gene transcription suggests that Cu^2+^ might disrupt the transcriptional regulatory network essential for thyroid differentiation and function, which could potentially contribute to the subsequent impairment of TH synthesis.

The *nis* and *tg* genes play essential roles in the synthesis of TH. Through the function of *nis*, iodide originating from the bloodstream is absorbed and concentrated within thyroid cells [36]. The expression of the *nis* gene is controlled by thyroid transcription factor (*ttf1* and *ttf2*) and *pax8*, and is activated under the stimulation of TSH [38]. In this study, the downregulation of *nis* was accompanied by the downregulation of *tshβ*, *ttf1,* and *pax8* genes after exposure to Cu^2+^ for 14 days. Therefore, the collective downregulation of *tshβ*, *ttf1*, and *pax8* by Cu^2+^ most likely led to the observed decrease in *nis* mRNA, implying a reduced ability of the thyroid gland to take up iodide. A previous study has demonstrated that TG is a dimeric protein synthesized and exclusively utilized within the thyroid gland, where it serves as a precursor for the production of THs. *Tg* expression exhibited TH sensitivity, serving as a potential biomarker for assessing thyroid function in standard development. In our study, *tg* gene expression was decreased following Cu^2+^ exposure. This finding aligns with the observed decrease in TH (T3 and T4) contents. In summary, Cu^2+^ exposure disrupted the expression of key genes of the HPT axis. This consistent downregulation of synthesis genes might explain the decrease in T3 and T4 levels. 

Following production, THs (mostly T4) attach to TTR for conveyance to responsive areas [39]. In this study, exposure to Cu^2+^ led to the downregulation of *ttr* gene expression alongside a reduction in T4 levels, which may be attributed to the decreased T4 content resulting from Cu^2+^ exposure.

We also analyzed the expression of deiodinase genes, which encode enzymes that modulate TH levels at the peripheral level. In fish, *dio2* catalyzes the deiodination of the inner ring of T4 to generate the active hormone T3 [22]. *Dio1* is generally considered to have a limited role in maintaining plasma TH homeostasis but plays a significant part in iodine recovery and TH degradation [40]. In this study, *dio1* expression was significantly upregulated in the Cu^2+^ exposure groups, which may contribute to the observed reduction in T3 levels. Furthermore, after 7 and 14 days of Cu^2+^ exposure, mRNA expression of *dio2* was significantly increased compared with the control group. The elevated transcription of *dio2* could partially explain the decreased circulating T4 levels and likely exerts a substantial effect on TH homeostasis within tissues [41]. A previous study also demonstrated that reduced whole-body T3 and T4 levels in juvenile grass carp were accompanied by increased expression of the *dio1* and *dio2* genes following Cd^2+^ exposure [42].

THs mediate their actions by binding to specific TRs, which function as ligand-activated transcription factors [22,43]. In this study, the expression of *trβ* was significantly upregulated in all Cu^2+^-exposed groups compared with the control, possibly reflecting an autoregulatory mechanism in response to reduced THs levels. In contrast to the reported downregulation in Chinese rare minnow [34], notable upregulation of *trβ* in *S. younghusbandi* was found. This inconsistency might be explained by the well-documented role of Cu^2+^ as a TR antagonist; it can suppress T3-induced responses [44] and compete for T3-binding sites [45]. Thus, the downregulation of *trβ* observed in the present study likely reflects a direct disruptive effect of Cu^2+^ on the thyroid system. It is important to note that the evidence for thyroid disruption presented in this study is based on hormonal assays and gene expression profiles. Further histological investigations of the thyroid gland are required to validate the observed thyroid-disrupting effects in the present study.

The thyroid-disrupting effects of Cu^2+^ observed in *S. younghusbandi* were not an isolated phenomenon but appeared to be a conserved toxicological response in fish. For instance, Cu^2+^ exposure led to a reduction in T3 levels and upregulation of *tshβ* in zebrafish [17]. Furthermore, a study on common carp demonstrated that Cu^2+^ exposure significantly elevated plasma T4 levels [46]. These studies confirmed that the HPT axis is a sensitive target for Cu^2+^ toxicity in fish. It is noteworthy that cuprous ions (Cu^+^) are known to form stable complexes with iodide, which could theoretically interfere with iodine uptake and thyroid hormone synthesis in a manner distinct from Cu^2+^. The present study focused on Cu^2+^, as it is commonly reported in environmental monitoring. Future investigations comparing the thyroid-disrupting effects of Cu^+^ and Cu^2+^, particularly regarding iodine metabolism, would be valuable to fully elucidate copper’s endocrine toxicity mechanisms.

Cu^2+^ could affect antioxidants in tissues and cause the generation of reactive oxygen species (ROS), resulting in severe peroxidative damage to cell membranes, DNA, and other macromolecules [47,48]. Antioxidant defense systems, found in all aerobic cells, could scavenge excess ROS and avoid oxidative damage to cells [49]. Among them, SOD and CAT serve as the primary line of defense against oxygen toxicity induced by metal exposure, functioning as key antioxidant enzymes. SOD facilitates the dismutation of the superoxide anion radical to water and hydrogen peroxide, which is detoxified by CAT activity [50]. We observed that the levels of SOD and CAT activities were significantly increased after exposure to 50 and 500 μg/L Cu^2+^. Previous studies have also shown that SOD and CAT activities in gills of rainbow trout, common carp, and gibel carp all showed a trend of induction after Cu^2+^ exposure [51]. This might be due to the increase in superoxide anions, and SOD and CAT activity were induced to maintain the dynamic balance between ROS removal and production in cells [52].

GSH has been reported to form GS-Me complexes with a range of metals via its thiolate sulfur group [53]. Specifically, GSH can reduce Cu^2+^ to form a stable Cu^+^-SG complex [54,55], thereby inhibiting further redox cycling and limiting the production of free radicals [56]. Therefore, the decrease in GSH content in larvae might be caused by higher concentrations and longer Cu^2+^ exposure. In addition, the GR enzyme is the critical enzyme of GSH. It plays a key role in regulating the oxidized GSH (GSSG)/GSH balance [57]. When its activity is enhanced, more GSSG is reduced back to GSH, thereby boosting the cell’s capacity to neutralize free radicals. In the present study, we observed that the activity of GR increased after Cu^2+^ exposure. It is speculated that these observed alterations may represent a compensatory mechanism in response to reduced GSH levels.

Lipid peroxidation is commonly recognized as a key indicator of oxidative stress in aquatic animals and is considered a major factor contributing to cellular dysfunction under oxidative stress conditions [58]. In this study, MDA, serving as a marker of oxidative stress, showed a significant increase in fish exposed to 50 and 500 μg/L of Cu^2+^ for 14 days, thus indicating the generation of serious oxidative stress. It is important to note that the assessment of oxidative stress relies on classical antioxidant markers. Further studies are needed to thoroughly understand the underlying mechanisms by directly measuring ROS and evaluating mitochondrial function.

The IBR is an effective method for quantitative evaluation of the toxic effects of pollutants on organisms and has been applied in many fields [59]. In our study, the IBR was applied to evaluate the changes in different biomarkers and summarize the subacute toxicity trend of Cu^2+^ exposure at different concentrations and for different durations. A star plot is used to present the parameters representing the reference deviation for each analyzed biomarker. Areas extending above 0 indicate biomarker induction, while those below 0 indicate biomarker inhibition. Moreover, the IBR combines the response of multiple biomarkers into a single value, providing an overall indication of the toxic stress level caused by Cu^2+^ exposure. The results of the IBR showed that *crh*, *dio1*, *dio2*, and *trβ*, the GR enzyme, and MDA contents of *S. younghusbandi* larvae were elevated, and *nis*, *tg*, *ttf*, *pax8*, and *ttr*, T4, and GSH contents were inhibited after exposure to Cu^2+^, which is consistent with the results of the toxicology experiments in this study. Additionally, with the increase in Cu^2+^ exposure concentration, the deviation in THs (T3 and T4), CAT, and MDA was more obvious. These appear to serve as biomarkers for monitoring Cu^2+^ pollution in *S. younghusbandi*. In the present study, we also observed that the calculated IBR values showed a strong correlation with both Cu^2+^ concentration and duration of exposure. These findings indicate that Cu^2+^ exposure exerts dose-dependent and time-dependent effects on *S. younghusbandi* larvae. As both the concentration of Cu^2+^ and the duration of exposure increase, *S. younghusbandi* larvae were subjected to more severe stress. Notably, while our higher exposure doses (50, 500 μg/L) defined toxicity thresholds, the IBR revealed a marked time-dependent effect even at the environmentally relevant level of 5 μg/L, the level at which the IBR value increased from 15.1 (7 days) to 23.6 (14 days). This indicates that prolonged exposure to Cu^2+^ concentrations within the reported environmental range could induce significant integrated stress, highlighting a potential chronic risk to *S. younghusbandi* larval development.

The IBR index has been proved to be a useful tool for integrating multi-level biomarker responses and visualizing the dose- and time-dependent stress induced by Cu^2+^. However, it is important to acknowledge the limitations of this approach. It inevitably oversimplifies the organism’s physiological state by reducing the complex and distinct biological effects to a single numerical value. Therefore, the IBR serves as a complementary tool that provides an overview of the overall stress and should be interpreted together with gene expression and hormone data to elucidate the specific toxicity mechanisms of Cu^2+^.

## 5. Conclusions

This study demonstrated that exposure to Cu^2+^ disrupted the thyroid endocrine system and induced oxidative stress in early life stages of *S. younghusbandi*, a dominant fish species in the ecologically sensitive Lhasa River. The key findings include a significant reduction in THs, dysregulation of genes critical for TH synthesis, transport, and metabolism, and impairment of the antioxidant enzyme system. These results indicate that native high-altitude fish are highly vulnerable to Cu^2+^ pollution. Moreover, the IBR results confirm that Cu^2+^ exposure exerted a dose- and time-dependent toxic effect, highlighting the risk even at lower concentrations under prolonged exposure. Future research should focus on validating the long-term and multi-generational toxicity of environmentally realistic Cu^2+^ levels to native fish.

## Figures and Tables

**Table 1:** The primer sequences used for qRT-PCR in this study.

Name	Sequence of Primer 5′-3′	Product Length (bp)	GenBank Accession
*β-actin-F*	GATGGACTCTGGTGATGGTGTGAC	167	NM_131031.2
*β-actin-R*	TTTCTCTTTCGGCTGTGGTGGTG		
*crh-F*	GATTTCCCTGGACCTGACC	131	XM_016522229.1
*crh-R*	GGGTTTGCTCGTGGTTACTT		
*tshβ-F*	GAGTTGGTGGGTCCTCGTT	177	NM_181494.2
*tshβ-R*	TTCTAAGGGCACATTCATCAC		
*ttf1-F*	AGAGCAAATGTGACCCAAGCAGAC	86	XM_016553336.1
*ttf1-R*	GGTTCTTCCTCGTGTTCGGTGATC		
*pax8-F*	GCAGTTCATCGCAGGCAGAGAC	94	XM_042757200.1
*pax8-R*	AGTGATGGTGGACGAGGAGAAGC		
*nis-F*	GACTATGCTGTATTTGCTGCTATG	102	XM_042729464.1
*nis-R*	GAAGAAGCTGTCCAGGTTAGAG		
*tg-F*	TGATGGTGCTGCGAAAGAAAGGTC	105	XM_016533797.1
*tg-R*	CAAGCGTCCGATACACTCCAGAAC		
*ttr-F*	TGGAGTTTGACACTAAAGCCTACT	110	XM_016518290.1
*ttr-R*	CCAGAGTGTAATGACGATGCC		
*dio1-F*	ATAAGCCAGCTGCCGATTT	152	XM_019086809.2
*dio1-R*	CTCCTCCAGATTACGGTGTTTC		
*dio2-F*	CAGATTTCCTGCTGGTCTACAT	134	XM_016567490.1
*dio2-R*	TCCTCCAGATTTCGGTGTTTC		
*trβ-F*	GAGGAGCAGCAGAATGAAAGA	130	XM_042735414.1
*trβ-R*	CCCTTGCGTTCACTCGATTA		

**Table 2:** Developmental indexes of *S. younghusbandi* larvae exposed to 0, 0.5, 5, 50, and 500 μg/L Cu^2+^ for 7 days and 14 days. Data are presented as mean ± SD (n = 3). Different lowercase letters denote significant differences among groups (*p* < 0.05).

Cu^2+^ (μg/L)	0	0.5	5	50	500
Malformation (7days, %)	1.33 ± 0.01 ^a^	1.47 ± 0.01 ^a^	1.91 ± 0.01 ^a^	2.86 ± 0.01 ^a^	3.12 ± 0.02 ^a^
Malformation (14 days, %)	2.02 ± 0.08 ^a^	2.79 ± 0.05 ^a^	4.26 ± 0.10 ^a,b^	5.77 ± 0.07 ^b^	6.92 ± 0.11 ^b^
Survival (7 days, %)	96.83 ± 0.01 ^a^	92.50 ± 2.16 ^a^	90.33 ± 1.25 ^a^	89.83 ± 1.24 ^a^	86.33 ± 2.36 ^a^
Survival (14 days, %)	92.83 ± 1.89 ^a^	85.33 ± 1.25 ^a^	80.17 ± 1.25 ^a,b^	77.33 ± 4.03 ^b^	68.83 ± 2.05 ^c^
Length (7 days, mm)	12.45 ± 0.31 ^a^	12.40 ± 0.36 ^a^	12.31 ± 0.18 ^a^	11.95 ± 0.18 ^a,b^	11.65 ± 0.49 ^b^
Length (14 days, mm)	13.84 ± 0.38 ^a^	13.52 ± 0.35 ^a^	13.51 ± 0.62 ^a^	13.08± 0.56 ^b^	12.46 ± 0.41 ^b^

## Data Availability

The original contributions presented in this study are included in the article/Supplementary Materials . Further inquiries can be directed to the corresponding author(s).

## Supplementary Materials

The following supporting information can be downloaded at: https://www.mdpi.com/article/10.3390/antiox15010112/s1, Table S1 . Summary of statistical tests applied to each variable.
