# Supplementary material for: Copper-Induced Thyroid Disruption and Oxidative Stress in Schizopygopsis younghusbandi Larvae
Source: Antioxidants (Basel). 2026 Jan 15;15(1):112. doi: 10.3390/antiox15010112 (PMC12838212; doi:10.3390/antiox15010112)
Supplement: Supplementary file 1 [file antioxidants-15-00112-s001.zip › antioxidants-4042096-supplementary.pdf]

Supplementary materials
For

Copper-Induced Thyroid Disruption and Oxidative Stress in *Schizopygopsis younghusbandi* Larvae

Liqiao Zhong [1,†], Chi Zhang [2,†], Fei Liu [2], Haitao Gao [3], Dengyan Di [4], Fan Yao [1,5,6], Baoshan Ma [1], Mingdian Liu [1,*] and Xinbin Duan [1,*]

[1] National Agricultural Science Observing and Experimental Station of Chongqing, Yangtze River Fisheries Research Institute, Chinese Academy of Fishery Sciences, Wuhan 430223, China; zhonglq@yfi.ac.cn (L.Z.); yaofann@stu.scu.edu.cn (F.Y.); baoshanma@yfi.ac.cn (B.M.)
[2] Institute of Fisheries Science, Xizang Academy of Agricultural and Animal Husbandry Sciences, Lhasa 850000, China; zc0891@163.com (C.Z.); liufei636@163.com (F.L.)
[3] Yunnan Academy of Fishery Sciences, Kunming 655000, China; ynkhtt@126.com
[4] Bureau of Agriculture and Rural Affairs of Gongshan County, Nujiang 673599, China; dengyandi22@163.com
[5] College of Fisheries, Huazhong Agricultural University, Wuhan 430070, China
[6] College of Water Resource & Hydropower, Sichuan University, Chengdu 610065, China
* Correspondence: liumd@yfi.ac.cn (M.L.); duan@yfi.ac.cn (X.D.); Tel.: +86-27-8178-0131 (M.L.); Fax: +86-27-8178-0088 (M.L.)
† These authors contributed equally to this work.

Table S1. Summary of statistical tests applied to each variable.

Dependent Variable	Time Point	Data Distribution / Transformation	Main Test	Post-hoc Test
Malformation rate	7 days	Parametric (Raw)	One-way ANOVA	Tukey's HSD
Malformation rate	14 days	Parametric (Raw)	One-way ANOVA	Tukey's HSD
Survival rate	7 days	Parametric (Raw)	One-way ANOVA	Tukey's HSD
Survival rate	14 days	Parametric (log-transformed)	One-way ANOVA	Tukey's HSD
Body length	7 days	Parametric (Raw)	One-way ANOVA	Tukey's HSD
Body length	14 days	Parametric (Raw)	One-way ANOVA	Tukey's HSD
T3 content	7 days	Non-parametric (Assumptions violated)	Kruskal-Wallis	Dunn's test
T3 content	14 days	Parametric (Raw)	One-way ANOVA	Tukey's HSD
T4 content	7 days	Parametric (log-transformed)	One-way ANOVA	Tukey's HSD
T4 content	14 days	Parametric (Raw)	One-way ANOVA	Tukey's HSD
CAT activity	7 days	Parametric (Raw)	One-way ANOVA	Tukey's HSD
CAT activity	14 days	Parametric (log-transformed)	One-way ANOVA	Tukey's HSD
SOD activity	7 days	Parametric (Raw)	One-way ANOVA	Tukey's HSD
SOD activity	14 days	Parametric (Raw)	One-way ANOVA	Tukey's HSD
GR activity	7 days	Parametric (Raw)	One-way ANOVA	Tukey's HSD
GR activity	14 days	Parametric (Raw)	One-way ANOVA	Tukey's HSD
GSH content	7 days	Parametric (Raw)	One-way ANOVA	Tukey's HSD
GSH content	14 days	Parametric (log-transformed)	One-way ANOVA	Tukey's HSD
MDA content	7 days	Parametric (Raw)	One-way ANOVA	Tukey's HSD
MDA content	14 days	Parametric (Raw)	One-way	Tukey's HSD

			ANOVA	
crh	7 days	Parametric (Raw)	One-way ANOVA	Tukey's HSD
crh	14 days	Parametric (Raw)	One-way ANOVA	Tukey's HSD
tshβ	7 days	Parametric (Raw)	One-way ANOVA	Tukey's HSD
tshβ	14 days	Parametric (Raw)	One-way ANOVA	Tukey's HSD
ttf1	7 days	Parametric (Raw)	One-way ANOVA	Tukey's HSD
ttf1	14 days	Parametric (Raw)	One-way ANOVA	Tukey's HSD
pax8	7 days	Parametric (Raw)	One-way ANOVA	Tukey's HSD
pax8	14 days	Parametric (Raw)	One-way ANOVA	Tukey's HSD
nis	7 days	Parametric (Raw)	One-way ANOVA	Tukey's HSD
nis	14 days	Parametric (Raw)	One-way ANOVA	Tukey's HSD
tg	7 days	Parametric (Raw)	One-way ANOVA	Tukey's HSD
tg	14 days	Parametric (Raw)	One-way ANOVA	Tukey's HSD
ttr	7 days	Parametric (Raw)	One-way ANOVA	Tukey's HSD
ttr	14 days	Parametric (Raw)	One-way ANOVA	Tukey's HSD
dio1	7 days	Parametric (Raw)	One-way ANOVA	Tukey's HSD
dio1	14 days	Parametric (log-transformed)	One-way ANOVA	Tukey's HSD
dio2	7 days	Parametric (Raw)	One-way ANOVA	Tukey's HSD
dio2	14 days	Parametric (log-transformed)	One-way ANOVA	Tukey's HSD
trβ	7 days	Parametric (log-transformed)	One-way ANOVA	Tukey's HSD
trβ	14 days	Parametric (log-transformed)	One-way ANOVA	Tukey's HSD
